# Challenges to a molecular approach to prey identification in the Burmese python, *Python molurus bivittatus*

**DOI:** 10.7717/peerj.1445

**Published:** 2015-11-24

**Authors:** Bryan G. Falk, Robert N. Reed

**Affiliations:** US Geological Survey, Fort Collins Science Center, Fort Collins, CO, USA

**Keywords:** Invasive species, Gut content analysis, PCR enrichment, Peptide nucleic acid clamp, Everglades, Prey, Predator, DNA barcoding

## Abstract

Molecular approaches to prey identification are increasingly useful in elucidating predator–prey relationships, and we aimed to investigate the feasibility of these methods to document the species identities of prey consumed by invasive Burmese pythons in Florida. We were particularly interested in the diet of young snakes, because visual identification of prey from this size class has proven difficult. We successfully extracted DNA from the gastrointestinal contents of 43 young pythons, as well as from several control samples, and attempted amplification of DNA mini-barcodes, a 130-bp region of *COX1*. Using a PNA clamp to exclude python DNA, we found that prey DNA was not present in sufficient quality for amplification of this locus in 86% of our samples. All samples from the GI tracts of young pythons contained only hair, and the six samples we were able to identify to species were hispid cotton rats. This suggests that young Burmese pythons prey predominantly on small mammals and that prey diversity among snakes of this size class is low. We discuss prolonged gastrointestinal transit times and extreme gastric breakdown as possible causes of DNA degradation that limit the success of a molecular approach to prey identification in Burmese pythons.

## Introduction

A major impact of an invasive predator species in its non-native range is the consumption of prey and concomitant decline of prey populations (Gurevitch & Padilla, 2004; Pimentel, Zuniga & Morrison, 2005; Zavaleta, Hobbs & Mooney, 2001). In the absence of direct predation observations, prey identity can be documented via visual or molecular analysis of predator gastrointestinal (GI) contents (i.e., material in the digestive tract; King et al., 2008; Symondson, 2002). A visual approach to prey identification is potentially problematic because: (1) it relies on specialized expert analysis; (2) sufficient identifiable material may not be present in the GI contents; and (3) a reference library for all life stages of all potential prey species may not be available (e.g., a predator may consume a young prey animal before durable, identifiable material such as exoskeletons, feathers, or hair have formed, or this material may have a different appearance during the prey species’ various life stages; Symondson, 2002). Not surprisingly, molecular approaches to prey identification are becoming more common because their costs are decreasing, they require minimal taxonomic expertise, and reference libraries of DNA sequences (e.g., GenBank) are becoming more complete (though libraries remain incomplete for many taxa; Kvist, 2013) (King et al., 2008; O’Rorke, Lavery & Jeffs, 2012; Symondson, 2002).

A DNA isolate from a sample of GI contents will almost certainly contain DNA from the predator in addition to the prey, and several enrichment methods are available to favor the sequencing of prey DNA (O’Rorke, Lavery & Jeffs, 2012; Pompanon et al., 2012). Two commonly employed enrichment methods for the sequencing of prey polymerase chain reaction (PCR) products are a blocking primer and a peptide nucleic acid (PNA) clamp (Pompanon et al., 2012). A blocking primer is a DNA oligomer that overlaps with the 3′ end of one of the PCR primers, is designed to anneal only to predator DNA sequences, and is modified so that it does not prime polymerization (i.e., it prevents amplification of predator DNA sequences by out-competing the PCR primers; Pompanon et al., 2012; Vestheim & Jarman, 2008). Similarly, a PNA clamp is a PNA oligomer that does not prime polymerization and is designed to anneal only to the predator sequences, so only the target locus of prey DNA is available for amplification (Chow et al., 2011; Pompanon et al., 2012). The PNA clamp is highly specific and has a relatively high melting temperature, making it generally more efficient at preventing predator DNA amplification than a blocking primer (Pompanon et al., 2012).

Species identification is an explicit goal of DNA barcoding, and in animals the DNA barcoding locus is typically an approximately 650-bp region of cytochrome *c* oxidase I (*COX1*; DeSalle, Egan & Siddall, 2005; Hebert, Cywinska & Ball, 2003). In some cases, the prey DNA contained in predator GI contents may be too degraded to amplify a locus of that size, making a smaller DNA fragment preferable (Hajibabaei et al., 2006). A shorter, 130-bp ‘mini-barcode’ region of *COX1* is sufficiently variable to identify most animals to species (Hajibabaei et al., 2006; Meusnier et al., 2008) and may be suitable for prey identification when DNA is degraded.

Our goal was to evaluate DNA barcoding as a tool for prey-species identification in invasive Burmese pythons (*Python molurus bivittatus*) in Florida. These snakes are known to consume at least 37 mammal and bird species (as well as the American alligator; Dove et al., 2011; Snow et al., 2007), and whereas the python population has been implicated in the decline of several species of meso-mammals in southern Florida (Dorcas et al., 2012; McCleery et al., 2015), the python’s impact on the majority of its prey species is unknown. For example, we do not know how prey selection is influenced by age and size of pythons, and this question is rendered more difficult because young pythons often consume young prey individuals that do not yet have adult feathers or hair; ontogenetic changes make reference libraries created from adult-stage morphologies potentially unsuitable (RW Snow, pers. comm., 2015). Development of an accurate DNA-based protocol for prey identification would facilitate further research into the impacts of Burmese pythons on the southern Florida ecosystem.

Our goal was to better understand the impact of the Burmese python on its prey populations in Florida, with two specific objectives related to molecular prey identification. First, we wanted to explore the utility of a molecular approach to prey identification in the Burmese python. Second, we wanted to characterize the diet of young Burmese pythons.

## Methods

We obtained live Burmese pythons from Everglades National Park via ongoing removal programs and humanely euthanized them using methods approved by the USGS Fort Collins Science Center Animal Care and Use Committee (FORT IACUC Approval 2015-02). Euthanized specimens were either placed on ice and necropsied within 24 h or frozen at −20 °C and thawed on ice at a later date for necropsy. Whenever GI contents were present, we noted their location (e.g., stomach) and stored the samples in plastic bags at −20 °C.

We selected GI-content samples from pythons <1.2 m in length for DNA extraction because we were interested in characterizing the diet of young pythons, and most or all pythons of this length are approximately ≤1 year in age (B Falk, 2015, unpublished data; see Supplemental Information 1). All these samples contained only hair (see ‘Results’), so we also selected three GI-content samples from larger pythons that contained only feathers so that we could evaluate DNA barcoding as a means to identify both mammalian and avian prey. Each frozen GI-content sample was thawed, subsampled, rinsed in water until just feathers and hair remained, and dried. There were no obvious differences in quality among these samples. We stored the cleaned and dried samples at −20 °C in sealed plastic sample bags, along with silica beads to keep them dry.

We extracted DNA from each sample using a Qiagen DNeasy Blood and Tissue Extraction Kit (Valencia, CA, USA), with two modifications to the manufacturer’s instructions. First, we added 10 µL of 1 M dithiothreitol (DTT) to each sample in the digestion step to facilitate the breakdown of feathers and hair (Campos & Gilbert, 2012). Second, we eluted with two volumes of 100 µL into two separate tubes (as opposed to 200 µL each) in the final step so that the DNA would not be too diluted. We also extracted DNA from the hair of a domestic housecat for a positive mammal control and from a dried blood sample of a Burmese python for a positive python control (we did not use DTT for the blood sample but otherwise followed the same extraction protocol for both of these control samples). We included feather bases (calami) in the feather extractions, and, though we could not identify hair follicles in the hair samples, we extracted from a sufficient quantity so that the follicles would be included if present.

We targeted the DNA mini-barcode for amplification using primers from Meusnier et al. (2008). The primers have already been used to amplify DNA from vertebrates, but we slightly modified them to better anneal to mammalian and avian sequences (Table 1; see Supplemental Information 1). We tested both a blocking primer and a PNA clamp as alternative approaches to avoid amplification of Burmese python DNA, and designed these using GenBank sequences of a Burmese python, an Indian python (*Python molurus molurus*), and eight bird and mammal species (see Supplemental Information 1). The 28-bp blocking primer overlaps with the forward barcoding primer and was modified by 3′ phosphorylation to prevent DNA polymerization (Table 1). This primer has a melting temperature approximately 3 °C higher than the barcoding primer. The 20-bp PNA clamp also overlaps with the forward barcoding primer and has a melting temperature approximately 19 °C higher than the barcoding primer (Table 1).

**Table 1:** Summary of primers and enrichment oligomers used to amplify prey DNA from Burmese python GI contents. The blocking primer and PNA clamp overlap with a 7- and 9-bp region, respectively, of the forward mini-barcoding primer.

Description	Name	Sequence (5′ → 3′)	*T_m_* (°C)	Size (bp)
Mini-barcoding primer—Forward	miniBarF	TCAACTAACCACAAAGATATYGGMAC	55.3	26
Mini-barcoding primer—Reverse	miniBarR	GAARATTATTACRAAWGCATGGGC	53.0	24
Blocking primer	coxBlock	TCGGCACATTATACCTACTATTTGGTGC/3Phos/	58.4	28
PNA clamp	coxPNA	TATCGGCACATTATACCTAC	74.0	20

We amplified all samples using Illustra PuReTaq Ready-To-Go™ PCR Beads (GE Healthcare, Pittsburg, PA, USA) in 25 µL reactions containing 2 µL DNA isolate and either 5 µL blocking primer, 5 µL PNA clamp, or no enrichment (see Supplemental Information 1 for PCR recipes and thermocycler programs). For a subset of eight samples (six GI contents, two positive controls), we attempted amplification using 10 µL DNA isolate to evaluate the effects of different starting template volumes. We re-amplified all PCR products in a second reaction using the PCR beads and just the barcoding primers (i.e., no enrichment) to search for low-quantity PCR products following the initial PCR. We visualized the amplicons on a 1% agarose gel stained with Sybr™ Safe (Life Technologies, Carlsbad, CA, USA) and cleaned them using 27 µL AMPure (Agencourt, Beverly, MA, USA) per 15 µL product. We prepared the sequencing reactions using BigDye v3.0 (Applied Biosystems, Foster City, CA, USA), cleaned them using ethanol precipitation, and sequenced them in both directions on an ABI3730xl (Applied Biosystems). Sequences were edited in Geneious v.5.4.6 (Biomatters, Auckland, New Zealand). We identified the sequences to species using the BLAST algorithm in GenBank (Sayers et al., 2011).

## Results

**Figure 1:**
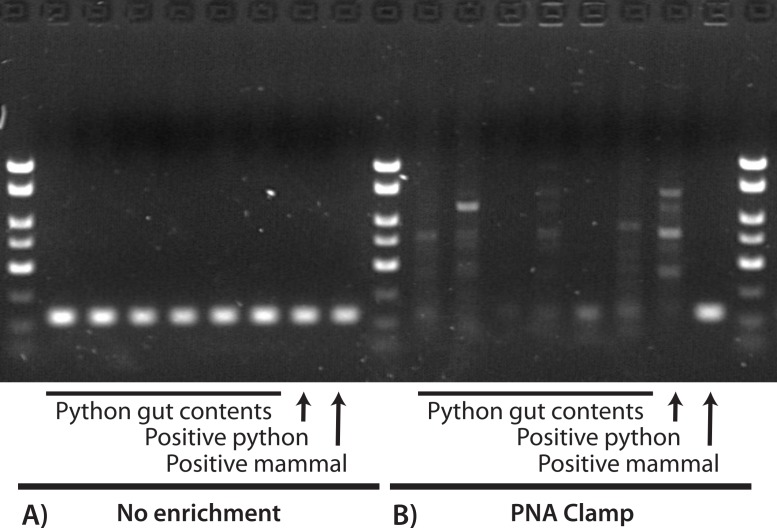
Visualization of PCR results for a 130-bp region of *COX1* with (A) no enrichment and (B) a PNA clamp to prevent amplification of python DNA. In both (A) and (B), the first six reactions used extractions from python GI contents, the seventh used a python positive control, and the eighth used a mammal positive control. We observed successful amplification of the target locus for all samples when no enrichment was used (A), and all sequences were identified as python except for the mammal positive control. When we prevented amplification of python DNA at the target locus (B), only the mammal positive control was successfully amplified. This suggests that: (1) our DNA extractions were successful; (2) the PNA clamp effectively excludes amplification of python DNA; and (3) prey DNA is of insufficient quality in these samples to amplify at this locus.

We identified and selected 43 GI-content samples from young pythons and three from adult pythons for DNA extraction. All 43 young-python samples contained only hair and all three adult samples contained only feathers. Of these combined 46 samples, 39 (89%) were collected from the large intestine and seven (11%) were collected from the stomach (see Supplemental Information 1). Using the barcoding primers and no enrichment we successfully amplified and sequenced the mini-barcode region from all 46 of these GI contents and the mammal and python positive controls. With the exception of the positive mammal control (which was correctly identified as cat), all of these sequences were identified as python. We also amplified and sequenced python DNA from a subset of six samples using the blocking primer, and so we abandoned the blocking primer as an enrichment approach. We did not sequence any python DNA when using the PNA clamp in the first PCR, recovering only the positive mammal control and one feather sample collected from the stomach that was identified (100% similarity) as limpkin, *Aramus guarauna*. In the second PCR (which used products from the first PCR as a template), we obtained satisfactory sequences of an additional six samples, all of which were identified (98% similarity) as the hispid cotton rat, *Sigmodon hispidus*. The remaining 39 samples produced either low-quality sequences that were identified as bacteria (i.e., non-target amplification; Siddall et al., 2009), low-quality and unidentifiable sequences, or no sequences at all. Note that these low-quality sequences were not mixed sequences of more than one prey species (e.g., clean chromatograms except multiple peaks at segregating sites). Figure 1 is a visual representation of these results for a subset of these reactions, where successful amplification of python DNA was observed for all GI-content samples when using only the barcoding primers, but only the mammal positive control was satisfactorily amplified when the PNA clamp was applied. We did not observe any differences between the PCR results that used 2 µL or 10 µL starting template.

We achieved slightly better success with the stomach-collected samples than intestine-collected samples. In addition to the sample amplified after the first PCR, one of the cotton-rat samples was also collected from the stomach, so that two of the seven (29%) of the successfully sequenced samples were collected from the stomach. In contrast, only four of 39 (11%) of the successfully sequenced samples were collected from the intestines. The success rate between stomach- and intestine-collected samples is not statistically different, however (chi-square = 0.512, *p* = 0.47).

## Discussion

Our results suggest that prey DNA in Burmese python GI contents is so degraded that a molecular approach to prey identification will only rarely be feasible. We successfully extracted DNA from all GI contents (we amplified/sequenced python DNA from all) but obtained prey sequence data from only 15% of the samples. In contrast, 82% and 95% of feather and mammal samples, respectively, in python GI contents were identifiable using morphology (though some of these were only identifiable to genus or family; Dove et al., 2011; Snow et al., 2007). We acknowledge that further efforts and technological advances may eventually make a molecular approach more successful, but we are skeptical that it will reach the efficiency of a visual approach in the near future.

The low quality of prey DNA in our samples may be a result of the natural digestive and behavioral characteristics of Burmese pythons, including efficient digestion, prolonged digestive times, and thermoregulation. The snakes consume their prey whole, and by the fourth day bones, skin, and other soft materials are no longer distinguishable (Secor, 2003). The digestive material leaves the stomach approximately one week after the meal, when only feathers or hair remain (Secor, 2003). Long digestion times are associated with degraded prey DNA (King et al., 2008), and Burmese pythons can retain their GI contents for several months, which is longer than many snakes and other predators (Lillywhite, De Delva & Noonan, 2002). Fluctuations in body temperate may further degrade prey DNA, because postprandial body temperatures in thermoregulating pythons rise above ambient and vary several degrees centigrade (Marcellini & Peters, 1982). It may be possible to increase the efficiency of a molecular approach for prey identification by limiting its use to stomach-collected samples—in which prey DNA has had less time to degrade—but only 10–11% of pythons removed from the greater Everglades ecosystem have prey in their stomachs (this study; Dove et al., 2011; Snow et al., 2007). In any case, additional hypothesis testing may provide insight into the reasons why prey DNA in Burmese python GI samples is so degraded and the potential solutions to these problems.

Other possible explanations for limited success in amplifying prey DNA include poor primer performance and blocking of prey DNA by the PNA clamp, but we consider these possibilities unlikely. In this study, we amplified snake, bird, and mammal sequences with our primers (this was done with great success in cases when we were not trying to amplify prey sequences), demonstrating their universality. PNA clamps are very unlikely to anneal to mismatched sequences (Pompanon et al., 2012), and the region we used appears conserved among pythons (it would also work in *Python regius*; GenBank AB177878) and not convergent with our wide taxonomic sample of mammalian and avian GenBank sequences.

Though limited, our results suggest that young Burmese pythons in Florida currently prey exclusively on small mammals. All the 43 young-python GI contents contained only hair, and the six of these from which we obtained prey DNA were identified as hispid cotton rat. These small native rodents are common in grassy areas of the southeastern United States (Bigler & Jenkins, 1975) and have previously been reported as Burmese python prey (Snow et al., 2007). In that study, 50 GI contents collected from 50 pythons averaging 265 cm in length were analyzed using visual analysis, and 18 prey species were reported, including 12 mammal, five bird, and one reptile species; cotton rats constituted approximately 10% of the total prey composition (Snow et al., 2007). A comparison with our results suggests that prey diversity may be relatively low in young Burmese pythons in the Everglades and that young pythons may be highly reliant on cotton rats. We have some understanding of the severe impacts of python predation on other mammal populations in Florida (e.g., marsh rabbit populations; McCleery et al., 2015), but the possible effects of python predation on the cotton rat and other small mammal populations, including direct impacts to the prey populations and indirect impacts such as reduced prey availability for native predator species, are unexplored.

## Supplemental Information

10.7717/peerj.1445/supp-1 Supplemental Information 1 Molecular ID of Burmese python prey—Supplementary Information (Falk and Reed)

## Additional Information and Declarations

### Data Availability

The following information was supplied regarding data availability: GenBank: KT992392–KT992398.
