# Supplementary material for: Challenges to a molecular approach to prey identification in the Burmese python, Python molurus bivittatus
Source: PeerJ. 2015 Nov 24;3:e1445. doi: 10.7717/peerj.1445 (PMC4662595; doi:10.7717/peerj.1445)
Supplement: Supplemental Information 1 [file peerj-03-1445-s001.docx]

Table S1. GenBank sequences used to design barcoding primers, blocking primer, and PNA clamp.

| Species | Common name | Taxon | GenBank number |
| --- | --- | --- | --- |
| *Python molurus bivattatus* | Burmese python | Reptile | KF010492 |
| *Python molurus molurus* | Indian python | Reptile | HM581978 |
| *Didelphis virginiana* | Virginia opossum | Mammal | NC_001610 |
| *Odocoileus virginianus* | White-tailed deer | Mammal | NC_015247 |
| *Procyon lotor* | Raccoon | Mammal | NC_009126 |
| *Rattus rattus* | Norway rat | Mammal | NC_012374 |
| *Aramus guarauna* | Limpkin | Bird | FJ027151 |
| *Coragyps atratus* | Black vulture | Bird | FJ027435 |
| *Mycteria americana* | Wood stork | Bird | JQ175426 |
| *Podilymbus podiceps* | Pie-billed grebe | Bird | DQ434722 |

Table S2. Collecting and specimen information for gut samples in our study.

| Sample ID | Collecting information | | | Total length  (cm) | Description (location) | Prey ID |
| --- | --- | --- | --- | --- | --- | --- |
|  | Date | Latitude | Longitude |  |  |  |
| 19Aug14.02 | 19-Aug-2014 | 25.62130 | -80.57556 | 59.0 | Hair (intestine) | Unable |
| 19Aug13.01 | 17-Aug-2013 | 25.60889 | -80.57510 | 67.0 | Hair (intestine) | Unable |
| 16Aug13.01 | 16-Aug-2013 | 25.63692 | -80.57616 | 70.0 | Hair (intestine) | Unable |
| 19Aug14.03 | 19-Aug-2014 | 25.62365 | -80.57563 | 71.5 | Hair (stomach) | Unable |
| 15Aug14.04 | 15-Aug-2014 | 25.63318 | -80.57597 | 72.0 | Hair (intestine) | Unable |
| 18Aug14.01 | 18-Aug-2014 | 25.60836 | -80.57505 | 72.5 | Hair (intestine) | Unable |
| 25Sep14.01 | 25-Sep-2014 | 25.59403 | -80.57457 | 72.5 | Hair (intestine) | Unable |
| 12Aug14.03 | 12-Aug-2014 | 25.60010 | -80.57478 | 73.5 | Hair (intestine) | Unable |
| 17Aug14.02 | 17-Aug-2014 | 25.61317 | -80.57528 | 74.5 | Hair (intestine) | Unable |
| 31Jul14.01 | 31-Jul-2014 | 25.60850 | -80.55003 | 75.5 | Hair (stomach) | Hispid cotton rat |
| 15Aug14.02 | 15-Aug-2014 | 25.62133 | -80.57555 | 76.0 | Hair (intestine) | Unable |
| 17Aug13.01 | 17-Aug-2013 | 25.62645 | -80.57577 | 76.5 | Hair (intestine) | Unable |
| 18Aug14.02 | 18-Aug-2014 | 25.64036 | -80.57622 | 78.0 | Hair (intestine) | Unable |
| 21Aug13.01 | 21-Aug-2013 | 25.62466 | -80.57562 | 78.0 | Hair (intestine) | Hispid cotton rat |
| 17Aug13.02 | 17-Aug-2013 | 25.60421 | -80.57494 | 78.5 | Hair (intestine) | Unable |
| 31Aug13.01 | 31-Aug-2013 | 25.56465 | -80.57510 | 79.0 | Hair (intestine) | Unable |
| 19Aug14.01 | 19-Aug-2014 | 25.62195 | -80.57556 | 79.5 | Hair (stomach) | Unable |
| 20Apr15.03 | 20-Apr-2015 | 25.41717 | -80.64945 | 79.5 | Hair (intestine) | Unable |
| 14Sep14.03 | 14-Sep-2014 | 25.57092 | -80.57515 | 80.0 | Hair (intestine) | Unable |
| 15Aug14.01 | 15-Aug-2014 | 25.64010 | -80.57625 | 80.0 | Hair (intestine) | Unable |
| 10Sep14.02 | 10-Sep-2014 | 25.62454 | -80.57570 | 80.5 | Hair (intestine) | Unable |
| 12Aug14.02 | 12-Aug-2014 | 25.62355 | -80.57565 | 80.5 | Hair (intestine) | Unable |
| 25Aug14.02 | 25-Aug-2014 | 25.60143 | -80.57485 | 81.0 | Hair (intestine) | Unable |
| 20Aug14.02 | 20-Aug-2014 | 25.61258 | -80.57527 | 82.5 | Hair (intestine) | Unable |
| 08Nov14.01 | 8-Nov-2014 | 25.09065 | 80.55165 | 83.0 | Hair (intestine) | Hispid cotton rat |
| 01Oct14.02 | 01-Oct-2014 | 25.58548 | -80.57518 | 84.0 | Hair (intestine) | Unable |
| 19Oct14.01 | 19-Oct-2014 | 25.60843 | -80.56162 | 84.5 | Hair (intestine) | Unable |
| 26Sep14.01 | 26-Sep-2014 | 25.60338 | -80.57487 | 90.0 | Hair (intestine) | Hispid cotton rat |
| 13Sep14.02 | 13-Sep-2014 | 25.60840 | -80.57213 | 92.0 | Hair (intestine) | Hispid cotton rat |
| 24Sep14.01 | 24-Sep-2014 | 25.60453 | -80.57493 | 93.0 | Hair (intestine) | Unable |
| 29Sep14.01 | 29-Sep-2014 | 25.62290 | -80.57565 | 93.0 | Hair (intestine) | Unable |
| 30Sep14.02 | 30-Sep-2014 | 25.59563 | -80.57460 | 93.5 | Hair (intestine) | Unable |
| 26Aug13.01 | 26-Aug-2013 | 25.60842 | -80.57495 | 94.0 | Hair (intestine) | Unable |
| 11Mar14.02 | 25-Dec-2013 | Unknown | Unknown | 96.0 | Hair (intestine) | Unable |
| 19Sep14.02 | 19-Sep-2014 | 25.56797 | -80.57515 | 97.0 | Hair (stomach) | Unable |
| 23Oct14.01 | 23-Oct-2014 | 25.59878 | -80.57473 | 99.5 | Hair (intestine) | Unable |
| 13Sep06.01 | 13-Sep-2006 | Unknown | Unknown | 103.5 | Hair (intestine) | Unable |
| 04Oct14.02 | 04-Oct-2014 | 25.59183 | -80.57517 | 108.0 | Hair (intestine) | Unable |
| 10Nov14.01 | 10-Nov-2014 | 25.57513 | -80.57522 | 108.0 | Hair (intestine) | Unable |
| 29Oct14.02 | 29-Oct-2014 | 25.60052 | -80.57483 | 110.0 | Hair (intestine) | Unable |
| 30Oct14.01 | 30-Oct-2014 | 25.60842 | -80.56065 | 110.0 | Hair (intestine) | Unable |
| 08May08.01 | 08-May-2008 | Unknown | Unknown | 111.5 | Hair (intestine) | Unable |
| 15Oct14.04 | 15-Oct-2014 | 25.59195 | -80.57520 | 114.0 | Hair (intestine) | Hispid cotton rat |
| 15Oct14.02 | 15-Oct-2014 | 25.59985 | -80.57478 | 119.5 | Hair (intestine) | Unable |
| 20Nov14.01 | 20-Nov-2014 | 25.59077 | -80.57522 | 119.5 | Hair (intestine) | Unable |
| 03Jan15.01 | 3-Jan-2015 | 25.43507 | 80.40341 | 215.0 | Feathers (stomach) | Unable |
| 01Jan15.01 | 1-Jan-2015 | 25.44677 | 80.40386 | 216.0 | Feathers (stomach) | Limpkin |
| 22Jan15.01 | 22-Jan-2015 | 25.23926 | -80.81206 | 265.0 | Feathers (intestine) | Unable |

Table S3. PCR recipes used to amplify prey DNA from Burmese python gut contents. All reactions used PuReTaq Ready-To-Go^TM^ PCR Beads. All oligomers are at a 10µM concentration, and all volumes are in µl.

| Description | Each mini-barcode primer | Enrichment | DNA Template | Water |
| --- | --- | --- | --- | --- |
| No enrichment | 0.5 | 0 | 2.0 | 22 |
| Second PCR | 0.5 | 0 | 1.0 | 24 |
| Blocking primer | 0.5 | 5.0 | 2.0 | 17 |
| PNA clamp | 0.5 | 5.0 | 2.0 | 17 |

Table S4. Thermocycler protocols used to amplify prey DNA from Burmese python gut contents. The ‘no enrichment’ protocol was used for initial PCRs when neither the blocking primer nor the PNA clamp were used, and for second, no-enrichment PCRs that used products from previous reactions (that did use a blocking primer or PNA clamp).

| Description | Initial melting | 40 cycles | | |  | Final Extension |
| --- | --- | --- | --- | --- | --- | --- |
|  |  | Melting | Annealing #1 | Annealing #2 | Extension |  |
| Blocking Primer | 2 min @ 94ºC | 30 sec @ 94ºC | 30 sec @ 53ºC | 30 sec @ 48ºC | 30 sec @ 72ºC | 5 min @ 72ºC |
| PNA Clamp | 2 min @ 94ºC | 30 sec @ 94ºC | 30 sec @ 58ºC | 30 sec @ 48ºC | 30 sec @ 72ºC | 5 min @ 72ºC |
| No enrichment | 2 min @ 94ºC | 30 sec @ 94ºC | 30 sec @ 48ºC | n/a | 30 sec @ 72ºC | 5 min @ 72ºC |
